# Genetic Characterization of *Brucella* spp.: Whole Genome Sequencing-Based Approach for the Determination of Multiple Locus Variable Number Tandem Repeat Profiles

**DOI:** 10.3389/fmicb.2021.740068

**Published:** 2021-11-12

**Authors:** Ana Pelerito, Alexandra Nunes, Teresa Grilo, Joana Isidro, Catarina Silva, Ana Cristina Ferreira, Sylvia Valdezate, Maria Sofia Núncio, Enrico Georgi, João Paulo Gomes

**Affiliations:** ^1^Emergency Response and Biopreparedness Unit, Department of Infectious Diseases, National Institute of Health, Lisbon, Portugal; ^2^Bioinformatics Unit, Department of Infectious Diseases, National Institute of Health, Lisbon, Portugal; ^3^CBIOS – Universidade Lusófona's Research Center for Biosciences & Health Technologies, Lisbon, Portugal; ^4^Faculty of Veterinary Medicine, Lusófona University, Lisbon, Portugal; ^5^Technology and Innovation Unit, Department of Human Genetics, National Institute of Health, Lisbon, Portugal; ^6^Centre for Toxicogenomics and Human Health (ToxOmics), Faculdade de Ciências Médicas, Nova Medical School, Universidade Nova de Lisboa, Lisbon, Portugal; ^7^National Institute for Agrarian and Veterinary Research, I.P. (INIAV, IP), Oeiras, Portugal; ^8^ISCIII Reference and Research Laboratory for Taxonomy, National Centre of Microbiology, Instituto de Salud Carlos III, Madrid, Spain; ^9^Bundeswehr Institute of Microbiology, Munich, Germany

**Keywords:** *Brucella* spp., MLVA, whole-genome sequencing, zoonosis, genotyping, Python script

## Abstract

Brucellosis is an important zoonosis that is emerging in some regions of the world, gaining increased relevance with the inclusion of the causing agent *Brucella* spp. in the class B bioterrorism group. Until now, multi-locus VNTR Analysis (MLVA) based on 16 loci has been considered as the gold standard for *Brucella* typing. However, this methodology is laborious, and, with the rampant release of *Brucella* genomes, the transition from the traditional MLVA to whole genome sequencing (WGS)-based typing is on course. Nevertheless, in order to avoid a disruptive transition with the loss of massive genetic data obtained throughout the last decade and considering that the transition timings will vary considerably among different countries, it is important to determine WGS-based MLVA alleles of the nowadays sequenced genomes. On this regard, we aimed to evaluate the performance of a Python script that had been previously developed for the rapid *in silico* extraction of the MLVA alleles, by comparing it to the PCR-based MLVA procedure over 83 strains from different *Brucella* species. The WGS-based MLVA approach detected 95.3% of all possible 1,328 hits (83 strains×16 loci) and showed an agreement rate with the PCR-based MLVA procedure of 96.4% for MLVA-16. According to our dataset, we suggest the use of a minimal depth of coverage of ~50x and a maximum number of ~200 contigs as guiding “boundaries” for the future application of the script. In conclusion, the evaluated script seems to be a very useful and robust tool for the *in silico* determination of MLVA profiles of *Brucella* strains, allowing retrospective and prospective molecular epidemiological studies, which are important for maintaining an active epidemiological surveillance of brucellosis.

## Introduction

Brucellosis is one of the most common bacterial zoonosis causing great damage to the farming industry and public health (Franc et al., 2018). The brucellosis burden specifically on low-income countries has led the World Health Organization (WHO) to classify it as one of the world’s leading neglected zoonotic diseases (WHO, 2020). However, given the absence of specific signs and symptoms, the disease is commonly underdiagnosed (Valdezate et al., 2010).

Brucellosis is transmitted to humans by ingestion of unpasteurized dairy products or by direct contact with infected animals, placentas, or aborted fetuses (Young, 2005). It can constitute a severely debilitating illness, with diverse symptoms ranging from fever, sweating, fatigue, weight loss, headache, and joint pain. Neurological complications, such as personality changes, meningitides, encephalitis, and peripheral neuropathy, can also occur (Dean et al., 2012).

The interest in human brucellosis has been boosted due to its recent re-emergence and enhanced surveillance worldwide and from the inclusion of the causing agent *Brucella* spp. in the group of class B bioterrorism agent (Franco et al., 2007). A low infectious dose of 100–1,000 organisms is sufficient to cause an infection. The mechanisms of transmission, through aerosols or food chains, make them easily transmissible to both humans and animals (Tan et al., 2015). Thus, the distinction between natural outbreaks and/or intentional release of microorganisms may be of fundamental importance in the context of the bioterrorism.

Studies enrolling DNA–DNA hybridization procedures and comparative genomics revealed that *Brucella* species are characterized by >80% interspecies homology and >98% sequence similarity (Whatmore et al., 2006; Kattar et al., 2008). Indeed, the sequencing of *16S rRNA* gene showed 100% identity between all *Brucella* spp. (Gee et al., 2004). The genus presently encloses 12 genetically highly related species. Human brucellosis can be caused by various *Brucella* species; however, *Brucella melitensis* is the most virulent and by far the most frequently observed causative agent of human infection (Young, 2005; Georgi et al., 2017). On this regard, the identification of the circulating *Brucella* species, biovar, and genotype is very important, mainly for tracking back infectious sources and monitoring transmission routes (Pisarenko et al., 2018). The species identification by PCR assays is sufficient for the purposes of diagnosis of human/animal disease or the detection of food contamination but not for the tracing of outbreaks or bioterrorism events (De Santis et al., 2011).

To achieve the goal of sub-species discrimination, Variable Number Tandem Repeats (VNTR) have been investigated in multi-locus VNTR analysis (MLVA) by various research groups since 2003 (Bricker et al., 2003; Le Fleche et al., 2006; Whatmore et al., 2006). This MLVA *Brucella* typing scheme has proved to have the ability to differentiate *Brucella* species, biovar, and even some isolates. This is facilitated do to the creation of an online database of MLVA-16 profiles (MLVA Bank, n.d.)^1^ that is available to all laboratories, allowing the comparison of *Brucella* strains worldwide (Le Fleche et al., 2006; Mambres et al., 2017; Sun et al., 2017). The recent implementation of whole genome single nucleotide polymorphism (SNP)-based typing, associated with its decreasing costs, has led to substantial improvements of both molecular subtyping and phylogenetic analyses in microbiology. The development of core- and whole- genome multilocus sequence typing (MLST) schemes has been focused on the restrict number of bacterial pathogens, including *Brucella* spp. but their application may be tricky (Tan et al., 2015; Janowicz et al., 2018; Sankarasubramanian et al., 2019). In fact, the creation of universal intra- or inter-species schemes needs to overcome some genetic hurdles such as the existence of paralogous genes, annotation issues, the accessory genome, and nomenclature-associated difficulties. Nevertheless, public databases for molecular typing and microbial genome diversity (PubMLST) are already available,^2^ allowing the use of whole genome sequences for typing purposes of multiple bacterial species. The same scenario is seen in the viral field as bioinformatics platforms were already developed, allowing the genotype determination from viral complete genomes, as for influenza virus and SARS-CoV-2 (Borges et al., 2018).^3^ Meanwhile, until whole genome data is fully established and accepted by the scientific community for classification/typing purposes in *Brucella*, the *in silico* determination of MLVA schemes can be of extreme utility. In fact, not only it overcomes the laborious PCR-based MLVA assessment but it also allows the dynamic cross-comparison with the typing-associated genetic data determined during the last decade. On this regard, a Python script has been developed focusing on the *in silico* determination of *Brucella* MLVA schemes taking advantage of the increasing number of sequenced genomes (Georgi et al., 2017). As no experimental validation of such script was performed, we now aimed to evaluate the agreement among the MLVA profiles determined through PCR- and WGS-based approaches for strains from several *Brucella* species, in order to check the validity of such technological transition underlying the genetic characterization of *Brucella*.

## Materials and Methods

### Samples

Eighty-three *Brucella* strains isolated in Portugal, Spain, Germany, Hungary, and Belgium were used in this study. This set comprises essentially not only *B. melitensis* but also some representatives of *B. suis*, *B. abortus*, and *B. ovis* (Supplementary Table S1). Unfortunately, we had no access to the isolates of low prevalent species such as *B. ceti*, *B. canis*, and *B. pinnipedialis*. *Brucella melitensis* 16M strain (NC_003317 and NC_003318) was used as a reference strain.

All samples were handled in a BSL-3 biocontainment laboratory at the Portuguese National Institute of Health. *Brucella* isolates were cultured on blood agar for 3–5days at 37°C under 5% CO_2_, and total DNA was extracted from fresh cultures on the NucliSens easyMAG platform (Biomerieux), according to the manufacturer’s instructions.

All strains were identified as *Brucella* species by real-time PCR, using a previously published assay (Pelerito et al., 2017). The molecular methods used up to species differentiation were performed in a tandem fashion. Firstly, an “in house” real-time PCR using hydrolysis probes was used to detect and identify *Brucella* genus. Secondly, for species differentiation, primers and Taqman probes were designed within the BMEII0466 gene for *B. melitensis* and BruAb2_0168 gene for *B. abortus* (Gopaul et al., 2008; Pelerito et al., 2017).

### MLVA Assays

Single locus amplification of the eight minisatelite loci (panel 1) and eight microsatelite loci (panels 2A and 2B), that constitute the MLVA-16 assay, was performed as describe by Le Fleche (Le Fleche et al., 2006), with modifications by Garofolo (2015). MLVA PCRs were performed in four multiplex reactions in a final volume of 10μl. The reactions contained: 1× Type-it Multiplex PCR Master Mix (Qiagen), 0.5× solution buffer, primers at appropriate concentrations, and 5μl of DNA. The thermocycling conditions were as follows: 96°C for 5min followed by either 30 (for multiplex 1, 3, and 4) or 24cycles (for multiplex 2) of: 95°C for 30s, 60°C for 90s, and 72°C for 30s; followed by 60°C for 30min. Multiplex 2 was run for 24cycles in order to contain VNTR amplification artifacts (Garofolo et al., 2013). A 1.1μl of each MLVA PCR product was mixed with 15μl of formamide-diluted GeneScan 500 LIZ dye or GeneScan 1,200 LIZ dye size standards (Applied Biosystems), depending on the expected size of the fragments, and denatured at 96°C for 3 or 5min, respectively. The mixtures were electrophoresed on an 8-capillary 3500 Genetic Analyzer equipped with 50cm-long capillaries and POP7 polymer (Applied Biosystems). Estimation of molecular sizes of PCR products was obtained using GeneMapper software 6 (Applied Biosystems) with default analysis parameters. The reference *B. melitensis* 16M strain, for which the expected size is known for each VNTR locus, was used as control for allele’s assignment.

### Whole Genome Sequencing

For each strain, WGS was performed as previously described (Pinto et al., 2018). Briefly, quantification and quality assessment of the purified DNA was performed using the DNA HS Assay Kit (Thermo Fisher Scientific) in the Qubit Fluorometer and agarose gel electrophoresis (0.8%), respectively. High-quality DNA samples were then used to prepare dual-indexed Nextera XT Illumina libraries that were subsequently subjected to cluster generation and paired-end sequencing (2×250bp and 2×300bp) on a MiSeq Illumina platform (Illumina Inc.), according to the manufacturer’s instructions.

Reads quality control and bacterial *de novo* assembly were performed using the INNUca v4.0.1 pipeline,^4^ which consists of several integrated modules for reads QA/QC, *de novo* assembly, and post-assembly optimization steps. Briefly, after reads’ quality analysis (FastQC v0.11.5)^5^ and cleaning (Trimmomatic v0.36; Bolger et al., 2014), genomes were assembled with SPAdes 3.11 (Bankevich et al., 2012) and subsequently improved using Pilon v1.18 (Walker et al., 2014), with genome coverage being monitored and reported after each processes. In order to evaluate the impact of the “post-assembly polishing” on the assembled genomes and subsequently on the *in silico* MLVA analyses, the SPAdes assemblies were also performed skipping the Pilon step. A final check was also performed. Considering that the *in silico* extraction of loci may be influenced by the quality of the assembled genomes, another largely used *de novo* assembler – Velvet (Zerbino and Birney, 2008) was applied through VelvetOptimiser v.2.2.5,^6^ for comparative purposes, with and without Pilon. The VelvetOptimiser script was run using trimmed reads for odd k-mer values ranging from 31 to 127 (highest k-mer used in SPAdes), with all program default settings unchanged apart from the minimum output contig size, which was the same as used by SPAdes.

### WGS-Based MLVA

Bacterial draft genomes were subjected to a Python script for *in silico* extraction of *Brucella* MLVA scheme (with 16 loci) as previously described (Georgi et al., 2017).^7^ This script is based on the count of repetitive DNA stretches contained within conserved DNA boxes that are upstream and downstream to the repetitions. As determining numbers of repeated stretches from WGS data may be error-prone, we carefully checked each locus in respect to the expected total length, internal repeat homogeneity or probability to get collapsed VNTRs during the assembly. All resulting MLVA 16 genotypes were compared to a public database with 2,215 entries of *B. melitensis* strains that can be assessed online (Grissa et al., 2008).^8^

### Accuracy Evaluation of the WGS-Based MLVA Approach

To assess the performance of the WGS-based MLVA approach, we determined the percentage of agreement between PCR- and WGS-based MLVA methods by calculating the number of identical results (i.e., identical called alleles), divided by the total number of hits that were detected simultaneously by both approaches. Although the PCR-based MLVA approach is considered as the gold standard (with obvious accuracy increment after the optimization of the multiplex PCR with fluorescent dyes for capillary electrophoresis), we believe the use of the “total number of hits that were detected simultaneously by both approaches” as the denominator, as the most reasonable and cautious procedure. In fact, although some hits were exclusively detected by the PCR-based MLVA approach, the opposite scenario was also observed. The maximum number of possible hits is 1,328 (i.e., 83 strains×16 loci).

Finally, for all strains, the performance of the bioinformatics script in extracting all MLVA loci was also evaluated by taking into account the quality of the draft genome generated by two assemblers (SPAdes and VelvetOptimiser) with and without “post-assembly polishing.” Briefly, for each condition, both the mean coverage depth and the number of contigs of each draft genome were correlated with the number of extracted alleles. Pearson’s coefficients (*r*) were measured to see potential linear associations. Nevertheless, as these final evaluations were done as complements of the major strategy, for the sake of clarity, whenever the text refers “WGS-based MLVA approach” it refers to the approach that used SPAdes with Pilon.

## Results

The performance of the WGS-based MLVA approach was assessed through the determination of the percentage of agreement with the results obtained by using the gold standard PCR-based MLVA. For the sake of clarity, we defined as “shared hits” the ones that were simultaneously detected by both approaches regardless their correct allele assignment. In this regard, the number of “matching alleles” was estimated using the number of “shared hits” as denominator.

**Figure 1:**
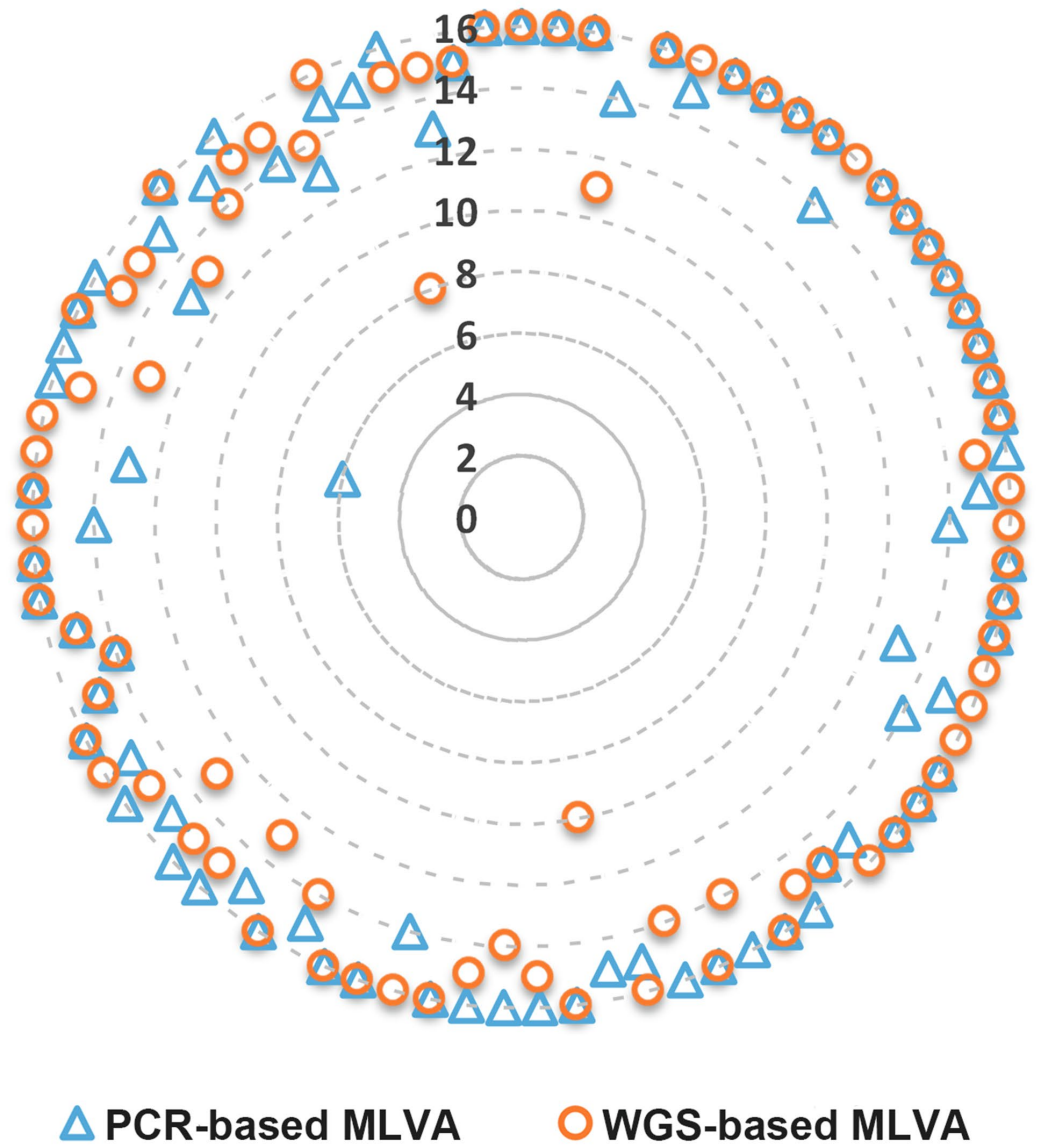
Number of MLVA loci detected per strain through PCR- and WGS-based MLVA approaches. The data for each approach are disposed along the imaginary radius of the circle graph (one radius *per* strain). The WGS-based results are relative to the MLVA loci extraction using draft genomes assembled with SPAdes 3.11 (Walker et al., 2014) and subsequently improved with Pilon v1.18 (Bankevich et al., 2012) from the INNUca v4.0.1 pipeline (see methods for details).

Overall, the WGS-based MLVA approach detected 1,265 of the 1,328 possible hits (95.3%). One, two, and ≥3 loci yielded no results for 18 (21.6%), 7 (8.4%), and 7 (8.4%) of the strains, respectively (Figure 1). Regarding the PCR-based MLVA approach, it detected 1,269 (95.6%) hits, whereas one, two, and ≥3 loci yielded no results for 18 (21.6%), 8 (9.6%), and 6 (7.2%) strains, respectively.

**Figure 2:**
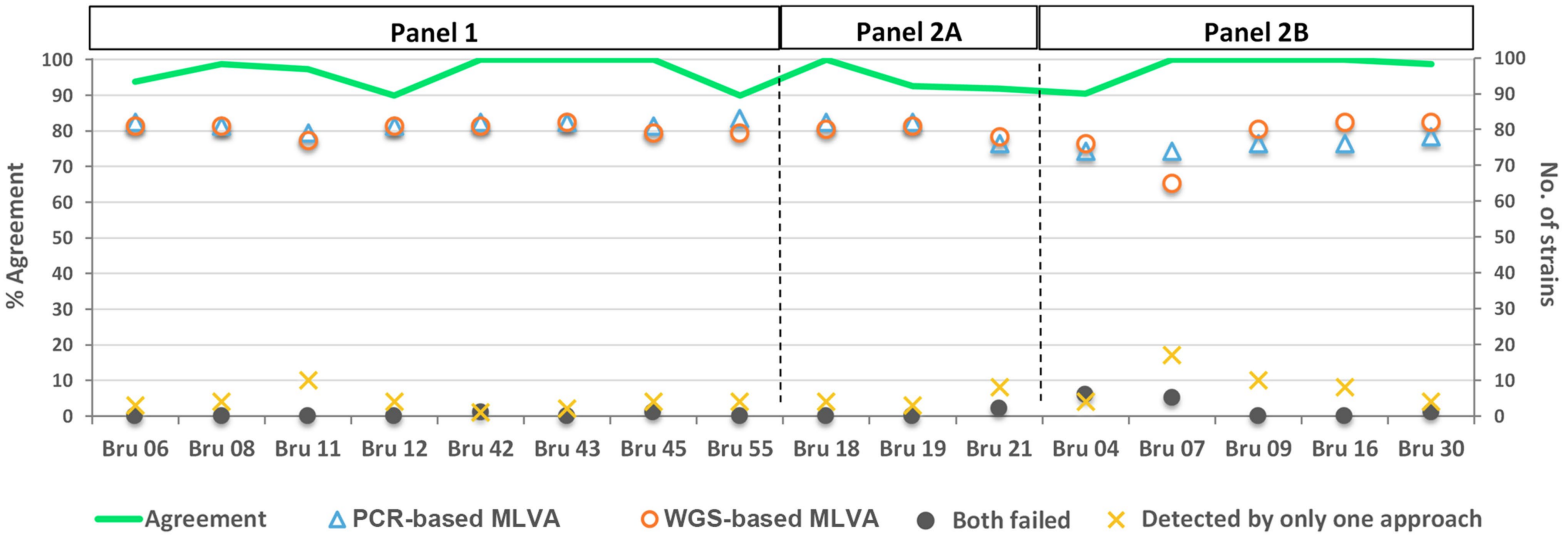
Performance of the PCR- and WGS-based MLVA approaches *per* locus. The graph represents the number of strains (right YY scale), for which it was possible to determine an allele *per* locus using each approach. The green line shows the percentage of agreement (left YY scale) *per* locus between both approaches (i.e., when identical alleles were called between the two methodologies). The loci are grouped according to the MLVA-16 panel they belong to (i.e., Panel 1, Panel 2A, and Panel 2B). The WGS-based results are relative to the MLVA loci extraction using draft genomes assembled with the INNUca v4.0.1 pipeline using Pilon v1.18 (Bankevich et al., 2012).

The allelic profiles obtained by both approaches are presented in Supplementary Table S2. Of the total 1,328 possible hits, 1,222 (92.0%) were simultaneously detected by both approaches (“shared hits”), whereas 6.8% were differently detected solely by one approach. These discrepancies were mostly observed for loci Bru07 and Bru09 (both from panel 2B) and Bru11 (from panel 1; Figure 2). Both MLVA approaches simultaneously failed the detection of 16 out of the 1,328 hits (1.2%), where 12 fall in panel 2B, specially focusing loci Bru04 and Bru07.

The analysis *per* locus showed an agreement rate of MLVA profiles (ratio of “matching alleles” *per* number of “shared hits”) determined through PCR- and WGS-based approaches ranging from 89.9 to 100.0% (Figure 2). The discrepancies involved loci from all three panels, being more pronounced in six loci (Bru06, Bru12, Bru55, Bru19, Bru21, and Bru04). In general, the mean of agreement of MLVA profiles for all 16 loci was 96.4%, revealing a high allele concordance between the two approaches.

**Figure 3:**
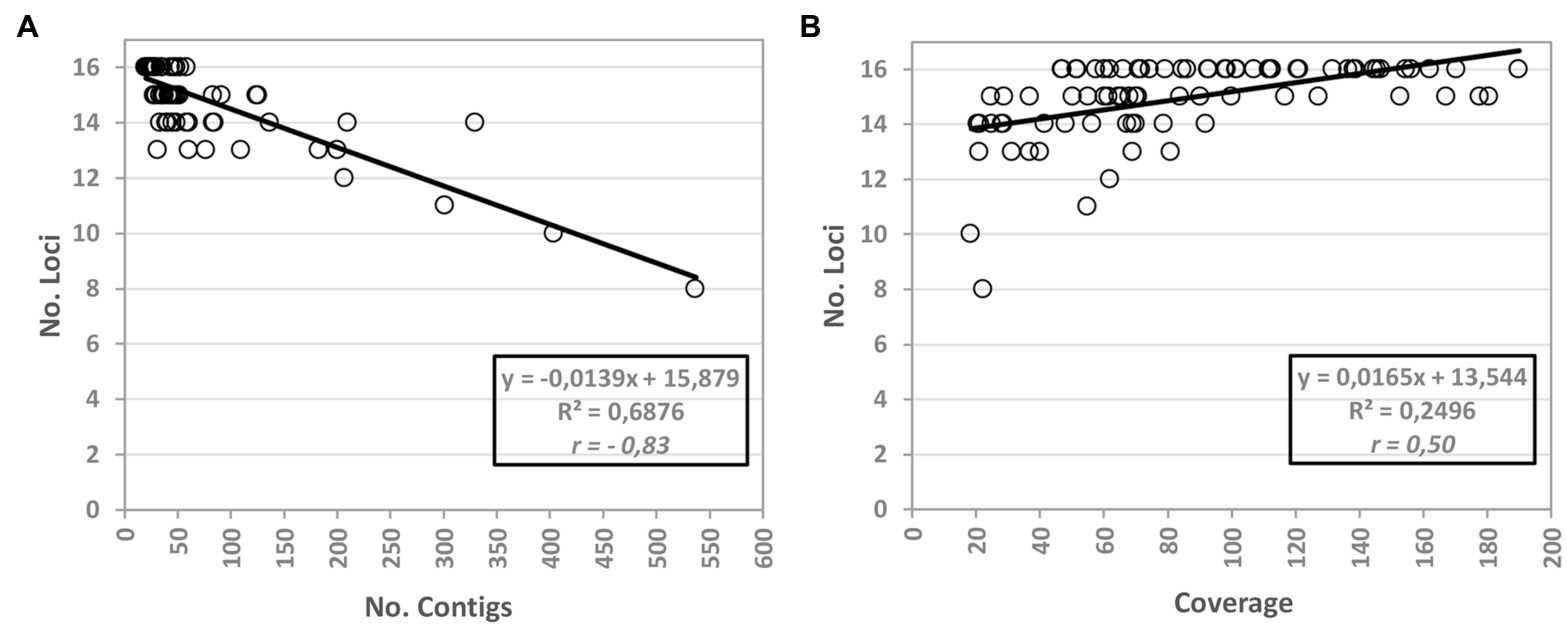
Influence of the mean coverage depth and number of contigs on the efficacy of the WGS-based MLVA extraction. The graphs show the correlation of the efficacy (measured by the number of loci for which an allele was called) of the bioinformatics script with the number of assembled contigs **(A)** and with the depth of coverage **(B)** after quality improvement. For (A,B), the tendency lines are shown with the respective equations and the Pearson coefficient (*r*). The WGS-based results are relative to the MLVA loci extraction using draft genomes assembled with the INNUca v4.0.1 pipeline using Pilon v1.18 (Bankevich et al., 2012).

We also inspected the quality of the draft sequences used as input because the Python script for *in silico* extraction of *Brucella* MLVA schemes is applied after the genome assembly and thus may be dependent on the quality of the “reads.” The influence of the mean depth of coverage and number of contigs on the efficacy of the bioinformatics script is illustrated in Figure 3. As expected, a negative linear correlation was observed among the efficacy of the *in silico* extraction and the number of assembled contigs, with less partitioned genomes allowing the detection of a higher number of alleles. On the other hand, higher genome mean coverage depth seem to favour the *in silico* extraction of MLVA loci.

As a final assessment, the performance of the bioinformatics script was also evaluated by using as input draft genome sequences assembled with different assemblers (SPAdes versus VelvetOptimiser; Supplementary Figure S1). Curiously, although no significant differences were observed regarding the number of loci extracted both with and without “post-assembly polishing” (data not shown), we observed that VelvetOptimiser was particularly affected by a low depth of coverage and genome fragmentation. In fact, for a mean depth of coverage <50 as well as for high fragmented genomes, the number of detected MLVA loci decreased more sharply when we used VelvetOptimiser than when SPAdes assemblies were used.

## Discussion

The control of brucellosis requires an accurate surveillance and the use of high discriminatory methods to characterize outbreak strains and determine the infection source and transmission routes. For many years, multiple typing methods were used for *Brucella* characterization at both species and biovar levels. These relied on host specificity, growth features, biochemical reactions, serotyping and bacteriophage typing, but they lacked discriminatory power (Sun et al., 2017). There are some studies focusing the application of WGS-based approaches to Brucella spp., where strains from multiple species and locations are commonly included (Pisarenko et al., 2018; Ashford et al., 2020; Esquivel et al., 2020; Pelerito et al., 2020). Nevertheless, currently, PCR-based MLVA is the most widely used approach for outbreak investigations and is still considered the gold standard for *Brucella* typing. Although the traditional MLVA assay relies on singleplex PCR followed by gel electrophoresis, some laboratories have already adopted a less error-prone approach based on multiplex PCRs and multicolor capillary electrophoresis (Garofolo, 2015; Vergnaud et al., 2018). The sixteen markers enrolled in the MLVA-16 scheme are a combination of moderately variable (minisatellites, panel 1) and highly discriminatory (microsatellites, panels 2A and 2B) *loci* (Al Dahouk et al., 2007). A MLVA typing assay depends on the selection of markers which individually would not provide a relevant clustering. Taken separately, the Tandem Repeat markers are either not informative enough, are too variable or show a high level of homoplasy. As such, the combination of well selected independent loci may be highly discriminatory as previously shown for other species (Le Fleche et al., 2006).

On behalf of the unavoidable transition from the classical typing to the WGS-based approaches, Python scripts were recently developed for the rapid *in silico* extraction of the *Brucella* MLVA alleles (Georgi et al., 2017; Vergnaud et al., 2018). This will allow the assignment of the MLVA types in the genomic era, avoiding the undesirable loss of genetic information that has been provided during more than 10years by using the gold standard PCR-based MLVA typing. This is also important because the timings for the technology transition will vary considerably among different countries. As no experimental evaluation had been performed so far for one of those scripts (Georgi et al., 2017), our main goal was to access its performance against the gold standard method.

Overall, the WGS-based MLVA approach detected 95.3% of all possible 1,328 hits and the agreement with the experimental method in detecting the correct alleles was found to be 96.4%. Despite this high level of agreement, some loci could not be detected either by both or by solely a single approach. Although most of the loci did not simultaneously fail for several strains, it is worth highlighting Bru04 and Bru07 (both from panel 2B), for which the PCR-based MLVA approach failed for nine out of the 83 strains under study. We may hypothesize the occurrence of experimental difficulties associated with a less optimized PCR for these specific loci. However, we observed that Bru04 and Bru07 were also the loci for which the WGS-based MLVA approach most failed (for seven and 18 strains, respectively). We speculate that this relies on the fact that these loci are among the ones with the shortest repetitive sequence stretches. This could hypothetically be an obstacle for a more precise experimental distinction between the number of repeats. Bioinformatically, short repetitive stretches may eventually impact MLVA loci determination mainly when high genome fragmentation occurs.

According to the results obtained for the present dataset, we observed that the performance of the WGS-based MLVA approach does not seem to be dependent on the post-assembly polishing but is clearly dependent on the depth of coverage and the degree of assembly fragmentation (where SPAdes performed better). Still, a minimal depth of coverage of ~50x and a maximum number of ~200 contigs (a range where both assemblers behaved similarly) seem to constitute guiding “boundaries” for the future application of the script. Of note, some failures underlying the WGS-based MLVA approach could hypothetically be solved by the long-reads technology as it usually overcomes the assembly problems associated with the repeated sequences, although this technology is more prone to yield sequencing errors. On the other hand, the use of very short reads (e.g., 100nt) may yield highly fragmented genomes and thus a more erroneous determination of the repeat number. In this study, we used the Illumina technology with reads of 250–300nt, which, together with a minimum depth of coverage of ~50x, yielded highly solid results.

In conclusion, although this study focused the most prevalent species (with emphasis in *B. melitensis*) the evaluated script seems to be a very useful and robust tool for *in silico* extraction of MLVA types of *Brucella* strains, dealing with a large number of samples in a short time period, and allowing retrospective and prospective molecular epidemiological studies. This allows a continuous and non-disruptive transition to a new typing era by putting the newly sequenced strains in the frame of the genetic characterization obtained for thousands of isolates collected worldwide throughout the last decade. This will certainly be important for public health reference laboratories to maintain an active epidemiological surveillance of brucellosis.

## Data Availability Statement

All raw sequence reads used in the present study were deposited in the European Nucleotide Archive under the study accession number PRJEB30030.

## Author Contributions

AP, AN, and JG conceived and designed the experiments, performed the analysis, interpreted the results, and wrote the manuscript. AP, JI, CS, AF, SV, and MN performed the experiments. EG performed the script optimization and implementation. All authors contributed to the article and approved the submitted version.

## Funding

This work is a result of the GenomePT project (POCI-01-0145-FEDER-022184), supported by the COMPETE 2020 – Operational Program for Competitiveness and Internationalisation (POCI), Lisboa Portugal Regional Operational Program (Lisboa2020), Algarve Portugal Regional Operational Program (CRESC Algarve2020), under the PORTUGAL 2020 Partnership Agreement, through the European Regional Development Fund (ERDF), and by the Fundação para a Ciência e a Tecnologia (FCT). The studies have arisen from the Project QUANDHIP (Chafea Grant Agreement no. 2010 21 02), which has been funded by the European Commission in the framework of the Health Program.

## Conflict of Interest

The authors declare that the research was conducted in the absence of any commercial or financial relationships that could be construed as a potential conflict of interest.

## Publisher’s Note

All claims expressed in this article are solely those of the authors and do not necessarily represent those of their affiliated organizations, or those of the publisher, the editors and the reviewers. Any product that may be evaluated in this article, or claim that may be made by its manufacturer, is not guaranteed or endorsed by the publisher.

## Footnotes

^1^ https://microbesgenotyping.i2bc.paris-saclay.fr

^2^ https://pubmlst.org/

^3^ https://insaflu.insa.pt/

^4^ https://github.com/B-UMMI/INNUca

^5^ http://www.bioinformatics.babraham.ac.uk/projects/fastqc/

^6^ https://github.com/tseemann/VelvetOptimiser

^7^ https://github.com/dinergate42/in-silico-typing

^8^ http://microbesgenotyping.i2bc.paris-saclay.fr/
