# Supplementary material for: Genetic Characterization of Brucella spp.: Whole Genome Sequencing-Based Approach for the Determination of Multiple Locus Variable Number Tandem Repeat Profiles
Source: Front Microbiol. 2021 Nov 12;12:740068. doi: 10.3389/fmicb.2021.740068 (PMC8633399; doi:10.3389/fmicb.2021.740068)
Supplement: Supplementary file 1 [file Image_1.pdf]

Supplementary Figure 1

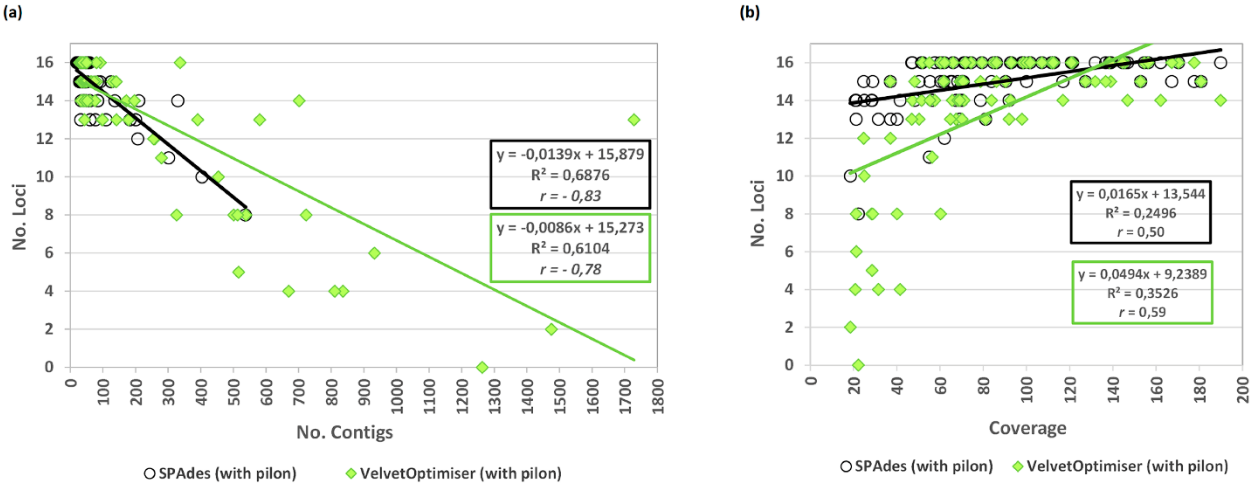

Supplementary Fig. S1 - Influence of the quality of the assembled genomes on the efficacy of the WGS–based MLVA loci extraction. The graphs show the correlation of the efficacy (measured by the number of loci for which an allele was called) of the bioinformatics script with the number of assembled contigs (panel (a)) and with the depth of coverage (panel (b)) after assembly polishing improvement. Two algorithms (SPAdes and VelvetOptimiser) were used to *de novo* assembly trimmed reads of each strain (see methods for details). The Pearson coefficients (*r*) are also shown for both graphs as well as the tendency lines shown with the respective equations.
