# Supplementary material for: Genetic Characterization of Brucella spp.: Whole Genome Sequencing-Based Approach for the Determination of Multiple Locus Variable Number Tandem Repeat Profiles
Source: Front Microbiol. 2021 Nov 12;12:740068. doi: 10.3389/fmicb.2021.740068 (PMC8633399; doi:10.3389/fmicb.2021.740068)
Supplement: Supplementary file 2 [file Table_1.DOCX]

| **Supplementary Table S1 - *Brucella* strains used in this study.** | | | |  |  |  |  |
| --- | --- | --- | --- | --- | --- | --- | --- |
| **Strain ID** | **Species** | **Isolation country** | **Isolation year** | **Host** | **BioSample Accession Number** | **Run Acession Number** | **Study** |
|  |  |  |  |  |  |  |  |
| MLVA01 | B. melitensis | Germany | Unknown | Unknown | ERS2983828 | ERR2993143 | this study |
| MLVA02 | B. melitensis | Germany | Unknown | Unknown | ERS2983829 | ERR2993144 | this study |
| MLVA03 | B. melitensis | Germany | Unknown | Unknown | ERS2983830 | ERR2993162 | this study |
| MLVA04 | B. melitensis | Germany | Unknown | Unknown | ERS2983831 | ERR2993153 | this study |
| MLVA05 | B. melitensis | Germany | Unknown | Unknown | ERS2983832 | ERR2993139 | this study |
| MLVA06 | B. melitensis | Germany | Unknown | Unknown | ERS2983833 | ERR2993160 | this study |
| MLVA07 | B. melitensis | Germany | Unknown | Unknown | ERS2983834 | ERR2993142 | this study |
| MLVA08 | B. melitensis | Germany | Unknown | Unknown | ERS2983835 | ERR2993136 | this study |
| MLVA09 | B. melitensis | Germany | Unknown | Unknown | ERS2983836 | ERR2993147 | this study |
| MLVA10 | B. melitensis | Germany | Unknown | Unknown | ERS2983837 | ERR2993149 | this study |
| MLVA11 | B. melitensis | Germany | Unknown | Unknown | ERS2983838 | ERR2993150 | this study |
| MLVA12 | B. melitensis | Germany | Unknown | Unknown | ERS2983839 | ERR2993161 | this study |
| MLVA13 | B. melitensis | Germany | Unknown | Unknown | ERS2983840 | ERR2993154 | this study |
| MLVA14 | B. melitensis | Germany | Unknown | Unknown | ERS2983841 | ERR2993133 | this study |
| MLVA16 | B. melitensis | Germany | Unknown | Unknown | ERS2983842 | ERR2993159 | this study |
| MLVA17 | B. melitensis | Germany | Unknown | Unknown | ERS2983843 | ERR2993141 | this study |
| MLVA18 | B. melitensis | Germany | Unknown | Unknown | ERS2983844 | ERR2993132 | this study |
| MLVA19 | B. melitensis | Germany | Unknown | Unknown | ERS2983845 | ERR2993146 | this study |
| MLVA20 | B. melitensis | Germany | Unknown | Unknown | ERS2983846 | ERR2993135 | this study |
| MLVA22 | Brucella spp. | Germany | Unknown | Unknown | ERS2983847 | ERR2993145 | this study |
| MLVA23 | Brucella spp. | Germany | Unknown | Unknown | ERS2983848 | ERR2993138 | this study |
| MLVA24 | Brucella spp. | Germany | Unknown | Unknown | ERS2983849 | ERR2993152 | this study |
| MLVA25 | Brucella spp. | Germany | Unknown | Unknown | ERS2983850 | ERR2993155 | this study |
| MLVA28 | B. melitensis | Germany | Unknown | Unknown | ERS2983851 | ERR2993137 | this study |
| MLVA29 | B. melitensis | Germany | Unknown | Unknown | ERS2983852 | ERR2993163 | this study |
| MLVA30 | B. melitensis | Germany | Unknown | Unknown | ERS2983853 | ERR2993151 | this study |
| MLVA31 | Brucella spp. | Germany | Unknown | Unknown | ERS2983854 | ERR2993140 | this study |
| MLVA32 | B. melitensis | Germany | Unknown | Unknown | ERS2983855 | ERR2993157 | this study |
| MLVA33 | B. melitensis | Germany | Unknown | Unknown | ERS2983856 | ERR2993148 | this study |
| MLVA34 | B. melitensis | Germany | Unknown | Unknown | ERS2983857 | ERR2993158 | this study |
| MLVA35 | Brucella spp. | Germany | Unknown | Unknown | ERS2983858 | ERR2993134 | this study |
| MLVA36 | B. melitensis | Germany | Unknown | Unknown | ERS2983859 | ERR2993156 | this study |
| MLVA37 | B. melitensis | Germany | Unknown | Unknown | ERS2983860 | ERR2993131 | this study |
| 120-99E | B. melitensis | Spain | 1999 | Homo sapiens | ERS2952734 | ERR2938672 | this study |
| 167-00E | B. melitensis | Spain | 2000 | Homo sapiens | ERS2952735 | ERR2938698 | this study |
| 170-04E | B. melitensis | Spain | 2004 | Homo sapiens | ERS2952736 | ERR2938644 | this study |
| 194-00E | B. melitensis | Spain | 2000 | Homo sapiens | ERS2952737 | ERR2938699 | this study |
| 204-01E | B. melitensis | Spain | 2001 | Homo sapiens | ERS2952738 | ERR2938692 | this study |
| 213-03E | B. melitensis | Spain | 2003 | Homo sapiens | ERS2952739 | ERR2938643 | this study |
| 228-03E | B. melitensis | Spain | 2003 | Homo sapiens | ERS2952740 | ERR2938694 | this study |
| 238-04E | B. melitensis | Spain | 2004 | Homo sapiens | ERS2952741 | ERR2938691 | this study |
| 297-04E | B. melitensis | Spain | 2004 | Homo sapiens | ERS2952742 | ERR2938671 | this study |
| 44-07E | B. melitensis | Spain | 2007 | Homo sapiens | ERS2952743 | ERR2938683 | this study |
| 457-06E | B. melitensis | Spain | 2006 | Homo sapiens | ERS2952744 | ERR2938657 | this study |
| 723-07E | B. melitensis | Spain | 2007 | Homo sapiens | ERS2952745 | ERR2938688 | this study |
| 104-11RK | B. melitensis | Germany | 2011 | Unknown | ERS2952746 | ERR2938681 | this study |
| 104-12RK | B. melitensis | Germany | 2012 | Unknown | ERS2952747 | ERR2938661 | this study |
| 104-13RK | B. melitensis | Germany | 2013 | Unknown | ERS2952748 | ERR2938652 | this study |
| 146-10RK | B. melitensis | Spain | 2010 | Unknown | ERS2952749 | ERR2938678 | this study |
| 146-12RK | B. melitensis | Spain | 2012 | Unknown | ERS2952750 | ERR2938648 | this study |
| 148-9RK | B. melitensis | Belgium | 2009 | Unknown | ERS2952751 | ERR2938665 | this study |
| 183-4RK | B. melitensis | Hungary | 2004 | Unknown | ERS2952752 | ERR2938701 | this study |
| 20Pa | B. melitensis | Portugal | 2002 | Goat | ERS2952753 | ERR2938658 | Pelerito, *et al.*, 2020 |
| 47Pa | B. melitensis | Portugal | 2001 | Sheep | ERS2952754 | ERR2938668 | Pelerito, *et al.*, 2020 |
| 357Pa | B. melitensis | Portugal | 2004 | Sheep | ERS2952755 | ERR2938653 | Pelerito, *et al.*, 2020 |
| 463Pa | B. melitensis | Portugal | 2005 | Sheep | ERS2952756 | ERR2938687 | Pelerito, *et al.*, 2020 |
| 770Pa | B. melitensis | Portugal | 2007 | Sheep | ERS2952757 | ERR2938669 | Pelerito, *et al.*, 2020 |
| 782Pa | B. melitensis | Portugal | 2007 | Goat | ERS2952758 | ERR2938670 | Pelerito, *et al.*, 2020 |
| 804Pa | B. melitensis | Portugal | 2008 | Bovine | ERS2952759 | ERR2938703 | Pelerito, *et al.*, 2020 |
| 918Pa | B. melitensis | Portugal | 2011 | Goat | ERS2952760 | ERR2938695 | Pelerito, *et al.*, 2020 |
| 1P | B. melitensis | Portugal | 2010 | Homo sapiens | ERS2952761 | ERR2938677 | Pelerito, *et al.*, 2020 |
| 35P | B. melitensis | Portugal | 2012 | Homo sapiens | ERS2952762 | ERR2938664 | Pelerito, *et al.*, 2020 |
| 36P | B. melitensis | Portugal | 2012 | Homo sapiens | ERS2952763 | ERR2938682 | Pelerito, *et al.*, 2020 |
| 38P | B. melitensis | Portugal | 2012 | Homo sapiens | ERS2952764 | ERR2938646 | Pelerito, *et al.*, 2020 |
| 40P | B. melitensis | Portugal | 2012 | Homo sapiens | ERS2952793 | ERR2938685 | Pelerito, *et al.*, 2020 |
| 41P | B. melitensis | Portugal | 2012 | Homo sapiens | ERS2952765 | ERR2938690 | Pelerito, *et al.*, 2020 |
| 43P | B. melitensis | Portugal | 2012 | Homo sapiens | ERS2952766 | ERR2938702 | Pelerito, *et al.*, 2020 |
| 44P | B. melitensis | Portugal | 2012 | Homo sapiens | ERS2952794 | ERR2938656 | Pelerito, *et al.*, 2020 |
| 66P | B. melitensis | Portugal | 2012 | Homo sapiens | ERS2952795 | ERR2938649 | Pelerito, *et al.*, 2020 |
| 147P | B. melitensis | Portugal | 2013 | Homo sapiens | ERS2952767 | ERR2938647 | Pelerito, *et al.*, 2020 |
| 165P | B. melitensis | Portugal | 2011 | Homo sapiens | ERS2952769 | ERR2938679 | Pelerito, *et al.*, 2020 |
| 166P | B. melitensis | Portugal | 2011 | Homo sapiens | ERS2952796 | ERR2938689 | Pelerito, *et al.*, 2020 |
| 167P | B. melitensis | Portugal | 2011 | Homo sapiens | ERS2952797 | ERR2938645 | Pelerito, *et al.*, 2020 |
| 168P | B. melitensis | Portugal | 2011 | Homo sapiens | ERS2952798 | ERR2938651 | Pelerito, *et al.*, 2020 |
| 169P | B. melitensis | Portugal | 2014 | Homo sapiens | ERS2952770 | ERR2938684 | Pelerito, *et al.*, 2020 |
| 183-7RK | B. ovis | Hungary | 2007 | Unknown | ERS2952784 | ERR2938660 | this study |
| 146-11RK | B. abortus | Spain | 2011 | Unknown | ERS2952785 | ERR2938686 | this study |
| 256Pa | B. abortus | Portugal | 2005 | Bovine | ERS2952786 | ERR2938666 | this study |
| 183-6RK | B. suis | Hungary | 2006 | Unknown | ERS2952787 | ERR2938662 | this study |
| 4Pa | B. suis | Portugal | 2000 | Swine | ERS2952788 | ERR2938697 | this study |
| 27Pa | B. suis | Portugal | 2003 | Swine | ERS2952789 | ERR2938673 | this study |
| 183Pa | B. suis | Portugal | 2009 | Boar | ERS2952791 | ERR2938650 | this study |
| 194Pa | B. suis | Portugal | 2011 | Swine | ERS2952792 | ERR2938675 | this study |
|  |  |  |  |  |  |  |  |
|  |  |  |  |  |  |  |  |
