# Supplementary material for: Genetic Characterization of Brucella spp.: Whole Genome Sequencing-Based Approach for the Determination of Multiple Locus Variable Number Tandem Repeat Profiles
Source: Front Microbiol. 2021 Nov 12;12:740068. doi: 10.3389/fmicb.2021.740068 (PMC8633399; doi:10.3389/fmicb.2021.740068)
Supplement: Supplementary file 3 [file Table_2.DOCX]

| **Supplementary Table S2 –** MLVA profile of PCR– and WGS–based approaches. | | | | | | | | | | | | | | | | | | | | | | |  |  |  |  |  |  |  |  |  |  |
| --- | --- | --- | --- | --- | --- | --- | --- | --- | --- | --- | --- | --- | --- | --- | --- | --- | --- | --- | --- | --- | --- | --- | --- | --- | --- | --- | --- | --- | --- | --- | --- | --- |
|  | **Bruce06** | | **Bruce08** | | **Bruce11** | | **Bruce12** | | **Bruce42** | | **Bruce43** | | **Bruce45** | | **Bruce55** | | **Bruce18** | | **Bruce19** | | **Bruce21** | | **Bruce04** | | **Bruce07** | | **Bruce09** | | **Bruce16** | | **Bruce30** | |
| **Strain** | **PCR - based MLVA** | **WGS - based MLVA** | **PCR - based MLVA** | **WGS - based MLVA** | **PCR - based MLVA** | **WGS - based MLVA** | **PCR - based MLVA** | **WGS - based MLVA** | **PCR - based MLVA** | **WGS - based MLVA** | **PCR - based MLVA** | **WGS - based MLVA** | **PCR - based MLVA** | **WGS - based MLVA** | **PCR - based MLVA** | **WGS - based MLVA** | **PCR - based MLVA** | **WGS - based MLVA** | **PCR - based MLVA** | **WGS - based MLVA** | **PCR - based MLVA** | **WGS - based MLVA** | **PCR - based MLVA** | **WGS - based MLVA** | **PCR - based MLVA** | **WGS - based MLVA** | **PCR - based MLVA** | **WGS - based MLVA** | **PCR - based MLVA** | **WGS - based MLVA** | **PCR - based MLVA** | **WGS - based MLVA** |
|  |  |  |  |  |  |  |  |  |  |  |  |  |  |  |  |  |  |  |  |  |  |  |  |  |  |  |  |  |  |  |  |  |
| ***Brucella abortus*** |  |  |  |  |  |  |  |  |  |  |  |  |  |  |  |  |  |  |  |  |  |  |  |  |  |  |  |  |  |  |  |  |
| **146-11RK** | 2 | 2 | 5 | 5 | ND | 4 | 11 | 11 | 2 | 2 | 2 | 2 | 3 | 3 | 3 | 3 | 6 | 6 | 43 | 43 | 8 | 8 | ND | ND | 5 | ND | 3 | 3 | 3 | 3 | ND | 5 |
| **256Pa** | 4 | 4 | 5 | 5 | 3 | 3 | 11 | 11 | 2 | 2 | 2 | 2 | 3 | 3 | 1 | 1 | 6 | 6 | ND | 43 | 8 | 8 | 4 | 4 | 6 | 6 | 7 | 7 | 3 | 3 | 3 | 3 |
| ***Brucella melitensis*** |  |  |  |  |  |  |  |  |  |  |  |  |  |  |  |  |  |  |  |  |  |  |  |  |  |  |  |  |  |  |  |  |
| **MLVA01** | 1 | 1 | 5 | 5 | 3 | 3 | 13 | 13 | 2 | 2 | 2 | 2 | 3 | 3 | 2 | 2 | 4 | 4 | 41 | 41 | 8 | 8 | 4 | 4 | 4 | 4 | 3 | 3 | 7 | 7 | 6 | 6 |
| **MLVA02** | 1 | 1 | 5 | 5 | 3 | 3 | 13 | 13 | 3 | 3 | 2 | 2 | 3 | 3 | 2 | 2 | 4 | 4 | 41 | 41 | 8 | 8 | 4 | 4 | 4 | 4 | 3 | 3 | 5 | 5 | 4 | 4 |
| **MLVA03** | 1 | 1 | 5 | 5 | 3 | 3 | 13 | 13 | 2 | 2 | 2 | 2 | 3 | 3 | 2 | 2 | 4 | 4 | 41 | 41 | 8 | 8 | 6 | 6 | 4 | 4 | 3 | 3 | 6 | 6 | 4 | 4 |
| **MLVA04** | 1 | 1 | 5 | ND | 3 | 3 | 13 | 13 | 2 | ND | 2 | 2 | 3 | 3 | 2 | ND | 4 | 4 | 41 | 41 | ND | ND | 5 | 5 | ND | ND | 3 | 3 | 5 | 5 | 4 | 4 |
| **MLVA05** | 1 | 1 | 5 | 5 | 3 | 3 | 13 | 13 | 3 | 3 | 2 | 2 | 3 | 3 | 2 | 2 | 4 | 4 | 42 | 41 | 8 | 8 | 4 | 4 | 4 | 4 | 3 | 3 | 6 | 6 | 4 | 4 |
| **MLVA06** | 1 | 1 | 5 | 5 | 3 | 3 | 13 | 13 | 3 | 3 | 2 | 2 | 3 | 3 | 2 | 2 | 4 | 4 | 41 | 41 | 7 | 8 | 4 | 4 | 4 | 4 | 3 | 3 | ND | 5 | 4 | 4 |
| **MLVA07** | 1 | 1 | 5 | 5 | 3 | 3 | 13 | 13 | 3 | 3 | 2 | 2 | 3 | 3 | 2 | 2 | 4 | 4 | 41 | 41 | 8 | 8 | 8 | 8 | 4 | 4 | 3 | 3 | 7 | 7 | 8 | 8 |
| **MLVA08** | 1 | 1 | 5 | 5 | 3 | 3 | 13 | 13 | 3 | 3 | 3 | 3 | 3 | 3 | 2 | 2 | 4 | 4 | 41 | 41 | 8 | 8 | 5 | 5 | 4 | 4 | 3 | 3 | 8 | 8 | 4 | 4 |
| **MLVA09** | 1 | 1 | 5 | 5 | 3 | 3 | 13 | 13 | 3 | 3 | 2 | 2 | 3 | 3 | 2 | 2 | 4 | 4 | 41 | 41 | 8 | 8 | 4 | 4 | 4 | 4 | 3 | 3 | 6 | 6 | 4 | 4 |
| **MLVA10** | 1 | 1 | 5 | 5 | 3 | 3 | 13 | 13 | 3 | 3 | 2 | 2 | 3 | 3 | 2 | 2 | 4 | 4 | 41 | 41 | 8 | 8 | 4 | 4 | 4 | 4 | 3 | 3 | 6 | 6 | 4 | 4 |
| **MLVA11** | 1 | 1 | 5 | 5 | ND | 3 | 13 | 13 | 3 | 3 | 2 | 2 | 3 | 3 | 2 | 2 | ND | 4 | 41 | 41 | 8 | 8 | 5 | 5 | 4 | 4 | 3 | 3 | 5 | 5 | 4 | 4 |
| **MLVA12** | 1 | 1 | 5 | 5 | 3 | 3 | 13 | 13 | 2 | 2 | 3 | 3 | 3 | 3 | 2 | 2 | 4 | 4 | 41 | 41 | 8 | 8 | 9 | 9 | 4 | 4 | 3 | 3 | 9 | 9 | 4 | 4 |
| **MLVA13** | 1 | 1 | 5 | 5 | 3 | 3 | 13 | 13 | 3 | 3 | 2 | 2 | 3 | 3 | 2 | 2 | 4 | 4 | 41 | 41 | 8 | 8 | 6 | 6 | 4 | 4 | 3 | 3 | 5 | 5 | 4 | 4 |
| **MLVA14** | 1 | 1 | 5 | 5 | 3 | 3 | 13 | 13 | 3 | 3 | 3 | 3 | 3 | 3 | 2 | 2 | 4 | 4 | 41 | 41 | 8 | 8 | 5 | 5 | 4 | 4 | 3 | 3 | 6 | 6 | 4 | 4 |
| **MLVA16** | 1 | 1 | 5 | 5 | 3 | 3 | 13 | 13 | 2 | 2 | 2 | 2 | 3 | 3 | 2 | 2 | 4 | 4 | 41 | 41 | 8 | 8 | 6 | 6 | 4 | 4 | 3 | 3 | 3 | 3 | 6 | 6 |
| **MLVA17** | 1 | 1 | 5 | 5 | 3 | 3 | 13 | 13 | 3 | 3 | 2 | 2 | 3 | 3 | 2 | 2 | 4 | 4 | 41 | 41 | 8 | 8 | 6 | 6 | 4 | 4 | 3 | 3 | 6 | 6 | 7 | 7 |
| **MLVA18** | 1 | 1 | 5 | 5 | 3 | 3 | 13 | 13 | 3 | 3 | 2 | 2 | 3 | 3 | 2 | 2 | 4 | 4 | 41 | 41 | 8 | 8 | 4 | 4 | 4 | 4 | 3 | 3 | 6 | 6 | 4 | 4 |
| **MLVA19** | 1 | 1 | 5 | 5 | 3 | 3 | 13 | 13 | 3 | 3 | 2 | 2 | 3 | 3 | 2 | 2 | 4 | 4 | 41 | 41 | 8 | 8 | 5 | 4 | 4 | 4 | 3 | 3 | 5 | 5 | 5 | 5 |
|  | **Bruce06** | | **Bruce08** | | **Bruce11** | | **Bruce12** | | **Bruce42** | | **Bruce43** | | **Bruce45** | | **Bruce55** | | **Bruce18** | | **Bruce19** | | **Bruce21** | | **Bruce04** | | **Bruce07** | | **Bruce09** | | **Bruce16** | | **Bruce30** | |
| **Strain** | **PCR - based MLVA** | **WGS - based MLVA** | **PCR - based MLVA** | **WGS - based MLVA** | **PCR - based MLVA** | **WGS - based MLVA** | **PCR - based MLVA** | **WGS - based MLVA** | **PCR - based MLVA** | **WGS - based MLVA** | **PCR - based MLVA** | **WGS - based MLVA** | **PCR - based MLVA** | **WGS - based MLVA** | **PCR - based MLVA** | **WGS - based MLVA** | **PCR - based MLVA** | **WGS - based MLVA** | **PCR - based MLVA** | **WGS - based MLVA** | **PCR - based MLVA** | **WGS - based MLVA** | **PCR - based MLVA** | **WGS - based MLVA** | **PCR - based MLVA** | **WGS - based MLVA** | **PCR - based MLVA** | **WGS - based MLVA** | **PCR - based MLVA** | **WGS - based MLVA** | **PCR - based MLVA** | **WGS - based MLVA** |
| **MLVA20** | 1 | 1 | 5 | 5 | 3 | 3 | 13 | 13 | 3 | 3 | 2 | 2 | 3 | 3 | 2 | 2 | 4 | 4 | 41 | 41 | 8 | 8 | 6 | 5 | 4 | 4 | 3 | 3 | 5 | 5 | 6 | 6 |
| **MLVA28** | 1 | 1 | 5 | 5 | 3 | 3 | 13 | 13 | 3 | 3 | 2 | 2 | 3 | 3 | 2 | 2 | 4 | 4 | 41 | 41 | 8 | 8 | 4 | 5 | 4 | 4 | 3 | 3 | 7 | 7 | 5 | 5 |
| **MLVA29** | 1 | 1 | 5 | 5 | 3 | 3 | 13 | 13 | 2 | 2 | 2 | 2 | 3 | 3 | 2 | 2 | 4 | 4 | 41 | 41 | 8 | 8 | 4 | 4 | 4 | 4 | 3 | 3 | 5 | 5 | 5 | 5 |
| **MLVA30** | 1 | 1 | 5 | 5 | ND | 3 | 13 | 13 | 3 | 3 | 2 | 2 | 3 | 3 | 2 | 2 | 4 | 4 | 41 | 41 | ND | 8 | 4 | 4 | 4 | 4 | 3 | 3 | ND | 8 | 5 | 5 |
| **MLVA32** | 1 | 1 | ND | 5 | 3 | 3 | ND | 14 | 2 | 2 | 4 | 4 | 3 | 3 | 2 | 2 | 4 | 4 | 41 | 41 | 8 | 8 | 6 | 5 | 4 | 4 | 3 | 3 | 6 | 6 | 6 | 6 |
| **MLVA33** | 1 | 1 | 5 | 5 | 3 | 3 | 13 | 13 | 2 | 2 | 2 | 2 | 3 | 3 | 2 | 2 | 4 | 4 | 41 | 41 | 8 | 8 | 6 | 6 | 6 | 6 | 3 | 3 | 5 | 5 | 5 | 5 |
| **MLVA34** | 1 | 1 | 5 | 5 | 3 | 3 | 13 | 13 | 2 | 2 | 3 | 3 | 3 | 3 | 2 | 2 | 4 | 4 | 41 | 41 | 8 | 8 | 6 | 6 | 4 | 4 | 3 | 3 | 6 | 6 | 4 | 4 |
| **MLVA36** | 1 | 1 | 5 | 5 | 3 | 3 | 13 | 13 | 3 | 3 | 2 | 2 | 3 | 3 | 2 | 2 | 4 | 4 | 46 | 45 | 9 | 8 | 8 | 8 | 4 | 4 | 3 | 3 | ND | 6 | 4 | 4 |
| **MLVA37** | 1 | 1 | 5 | 5 | 3 | 3 | 13 | 13 | 3 | 3 | 2 | 2 | 3 | 3 | 2 | 2 | 4 | 4 | 41 | 41 | 8 | ND | 6 | 6 | 4 | 4 | 3 | 3 | ND | 7 | 5 | 5 |
| **120-99E** | 1 | 1 | 5 | 5 | 3 | 3 | 13 | 13 | 2 | 2 | 2 | 2 | 3 | 3 | 2 | 2 | 4 | 4 | 41 | 41 | 8 | 8 | 7 | 7 | 7 | ND | 3 | 3 | 4 | 4 | 5 | 5 |
| **167-00E** | 3 | 3 | 4 | 4 | 3 | 3 | 13 | 13 | 4 | 4 | 2 | 2 | 3 | 3 | 3 | 3 | 4 | 4 | 36 | 36 | 6 | 5 | 2 | 2 | 5 | 5 | 3 | 3 | 4 | 4 | 5 | 5 |
| **170-04E** | 3 | 3 | 4 | 4 | 3 | 3 | 13 | 13 | 4 | 4 | 3 | 3 | 3 | 3 | 3 | ND | 6 | 6 | 36 | 36 | 6 | 5 | 2 | 2 | 5 | 5 | 6 | ND | 6 | 6 | 4 | 4 |
| **194-00E** | 1 | 1 | 5 | 5 | 3 | 3 | 13 | 13 | 2 | 2 | 2 | 2 | 3 | 3 | 2 | 2 | 4 | 4 | 41 | 41 | 8 | 8 | 7 | 7 | 7 | 7 | 3 | 3 | 4 | 4 | 6 | 6 |
| **204-01E** | 1 | 1 | 5 | 5 | 3 | 3 | 13 | 13 | 2 | 2 | 2 | 2 | 3 | 3 | 2 | 2 | 4 | ND | 41 | 41 | 8 | 8 | 5 | 5 | 7 | ND | 3 | 3 | 6 | 6 | 5 | 5 |
| **213-03E** | 3 | 3 | 4 | 4 | 2 | 2 | 13 | 13 | 3 | 3 | 2 | 2 | 3 | 3 | 3 | 3 | 8 | 8 | 36 | 36 | 6 | 6 | 2 | 2 | 4 | 4 | 6 | 6 | 3 | 3 | ND | 6 |
| **228-03E** | 1 | 1 | 5 | 5 | 3 | ND | 13 | 13 | 2 | 2 | 2 | 2 | 3 | ND | 2 | ND | 4 | 4 | 41 | 41 | 8 | 8 | 7 | ND | 7 | ND | 3 | 3 | 4 | 4 | ND | ND |
| **238-04E** | 1 | 1 | 5 | 5 | 3 | 3 | 13 | 13 | 2 | 2 | 2 | 2 | 3 | 3 | 2 | 2 | 4 | 4 | 41 | 41 | 8 | 8 | 5 | 7 | 5 | 5 | 3 | 3 | 4 | 4 | 6 | 6 |
| **297-04E** | 1 | 1 | 5 | 5 | 3 | 3 | 13 | 13 | 2 | 2 | 2 | 2 | 3 | 3 | 2 | 2 | 4 | 4 | 38 | 41 | 8 | 8 | 6 | 6 | 7 | ND | 3 | 3 | 4 | 4 | 5 | 5 |
| **44-07E** | 1 | 1 | 5 | 5 | 3 | 3 | 13 | 13 | 2 | 2 | 2 | 2 | 3 | 3 | 2 | 2 | 4 | 4 | 41 | ND | 8 | ND | 4 | 4 | 7 | 7 | 3 | 3 | 5 | 5 | 5 | 5 |
| **457-06E** | 1 | 1 | 5 | 5 | 3 | 3 | 13 | 13 | 2 | 2 | 2 | 2 | 3 | 3 | 2 | 2 | 4 | 4 | 41 | 41 | 8 | 8 | 5 | 5 | 7 | ND | 3 | 3 | 6 | 6 | 5 | 5 |
| **723-07E** | 1 | 1 | 5 | 5 | 3 | 3 | 13 | 13 | 2 | 2 | 2 | 2 | 3 | 3 | 2 | 2 | 4 | 4 | 41 | 41 | 8 | 8 | 5 | 5 | 6 | 6 | 3 | 3 | 6 | 6 | 5 | 5 |
| **104-11RK** | 2 | 3 | 4 | 4 | 3 | 3 | 13 | 13 | 4 | 4 | 3 | 3 | 3 | 3 | 3 | 3 | 4 | 4 | 36 | 36 | 5 | 5 | 2 | 2 | ND | 5 | 3 | 3 | ND | 4 | 5 | 5 |
| **104-12RK** | 1 | 1 | 5 | 5 | 3 | 3 | 13 | 13 | 2 | 2 | 2 | 2 | 3 | 3 | 2 | 2 | 4 | 4 | 41 | 41 | 8 | 8 | 8 | 8 | 7 | 7 | 3 | 3 | 7 | 7 | 5 | 5 |
| **104-13RK** | 1 | 1 | 5 | 5 | 3 | 3 | 13 | 13 | 2 | 2 | 2 | 2 | 3 | 3 | 2 | 2 | 4 | 4 | 41 | 41 | 8 | 8 | 4 | 4 | 7 | 7 | 3 | 3 | 5 | 5 | 5 | 5 |
| **146-12RK** | 2 | 2 | 4 | 4 | 3 | ND | 13 | 13 | 5 | 5 | 2 | 2 | 3 | 3 | 3 | 3 | 6 | 6 | 36 | 36 | 5 | 5 | 2 | 2 | ND | ND | 10 | 10 | 6 | 6 | 5 | 5 |
| **148-9RK** | 2 | 2 | 5 | 5 | 3 | 3 | 13 | 13 | 3 | 3 | 2 | 2 | 3 | 3 | 5 | 5 | 5 | 5 | 36 | 36 | 8 | 8 | 4 | 4 | 5 | 5 | 7 | 7 | 8 | 8 | 5 | 5 |
| **183-4RK** | 2 | ND | 3 | ND | 3 | ND | 10 | 13 | 3 | 3 | 1 | 1 | 3 | 3 | 2 | 2 | 4 | 4 | 38 | 38 | 9 | 9 | 2 | 2 | 3 | 3 | ND | 10 | 5 | 5 | 3 | 3 |
|  | **Bruce06** | | **Bruce08** | | **Bruce11** | | **Bruce12** | | **Bruce42** | | **Bruce43** | | **Bruce45** | | **Bruce55** | | **Bruce18** | | **Bruce19** | | **Bruce21** | | **Bruce04** | | **Bruce07** | | **Bruce09** | | **Bruce16** | | **Bruce30** | |
| **Strain** | **PCR - based MLVA** | **WGS - based MLVA** | **PCR - based MLVA** | **WGS - based MLVA** | **PCR - based MLVA** | **WGS - based MLVA** | **PCR - based MLVA** | **WGS - based MLVA** | **PCR - based MLVA** | **WGS - based MLVA** | **PCR - based MLVA** | **WGS - based MLVA** | **PCR - based MLVA** | **WGS - based MLVA** | **PCR - based MLVA** | **WGS - based MLVA** | **PCR - based MLVA** | **WGS - based MLVA** | **PCR - based MLVA** | **WGS - based MLVA** | **PCR - based MLVA** | **WGS - based MLVA** | **PCR - based MLVA** | **WGS - based MLVA** | **PCR - based MLVA** | **WGS - based MLVA** | **PCR - based MLVA** | **WGS - based MLVA** | **PCR - based MLVA** | **WGS - based MLVA** | **PCR - based MLVA** | **WGS - based MLVA** |
| **1P** | 1 | 1 | 5 | 5 | 3 | 3 | 13 | 13 | 2 | 2 | 2 | 2 | 3 | 3 | 2 | 2 | 4 | 4 | 41 | 41 | 8 | 8 | 6 | 6 | 7 | ND | 3 | 3 | 5 | 5 | 4 | 4 |
| **35P** | 1 | 1 | 5 | 5 | 3 | 3 | 13 | 13 | 2 | 2 | 2 | 2 | 3 | 3 | 2 | 2 | 4 | 4 | 41 | 41 | 8 | 8 | 6 | 6 | 6 | ND | 3 | 3 | 4 | 4 | 5 | 5 |
| **36P** | 1 | 1 | 5 | 5 | 3 | 3 | 13 | 13 | 2 | 2 | 2 | 2 | 3 | 3 | 2 | 2 | 4 | 4 | 41 | ND | 8 | ND | 6 | 6 | 7 | ND | 3 | 3 | 4 | 4 | ND | 5 |
| **38P** | 1 | 1 | 5 | 5 | 3 | 3 | 13 | 13 | 2 | 2 | 2 | 2 | 3 | 3 | 5 | 2 | 4 | 4 | 41 | 41 | 8 | 8 | 6 | 6 | 7 | ND | 3 | 3 | 4 | 4 | 5 | 5 |
| **40P** | 3 | 3 | 4 | 4 | 3 | 3 | 14 | 14 | 4 | 4 | 2 | 2 | 3 | 3 | 5 | 3 | 6 | 6 | 36 | 36 | 5 | 5 | 2 | 2 | 5 | 5 | 7 | 7 | ND | 3 | 5 | 5 |
| **41P** | 1 | 1 | 5 | 5 | 3 | 3 | 13 | 13 | 2 | 2 | 2 | 2 | 3 | 3 | 2 | 2 | 4 | 4 | 41 | 41 | 8 | 8 | 6 | 6 | 7 | 7 | 3 | 3 | 4 | 4 | 5 | 5 |
| **43P** | 1 | 1 | 5 | 5 | 3 | 3 | 13 | 13 | 2 | 2 | 2 | 2 | ND | ND | 5 | 2 | 4 | 4 | 41 | 41 | 8 | 8 | 5 | 5 | 7 | 7 | 3 | 3 | 4 | 4 | 5 | 5 |
| **44P** | 1 | 1 | 5 | 5 | 3 | 3 | 13 | 13 | 2 | 2 | 2 | 2 | 3 | 3 | 5 | 2 | 4 | 4 | 41 | 41 | ND | ND | 6 | 6 | ND | ND | 3 | 3 | 4 | 4 | 5 | 5 |
| **66P** | 1 | 1 | 5 | 5 | 3 | 3 | 13 | 13 | 2 | 2 | 2 | 2 | 3 | 3 | 2 | 2 | 4 | 4 | 41 | 41 | 8 | 8 | 7 | 7 | ND | ND | 3 | 3 | 6 | 6 | 5 | 5 |
| **147P** | 3 | 3 | 4 | 4 | 3 | 3 | 13 | 13 | 5 | 5 | 2 | 2 | 3 | 3 | 3 | 3 | 6 | 6 | 36 | 36 | 5 | 5 | 2 | 2 | 8 | 8 | 6 | 6 | 3 | 3 | 5 | 5 |
| **165P** | 1 | 1 | 5 | 5 | 3 | 3 | 13 | 13 | 3 | 3 | 2 | 2 | 3 | 3 | 2 | 2 | 5 | 5 | 7 | 9 | 8 | 8 | 3 | 3 | 4 | 4 | 3 | 3 | 4 | 4 | 6 | 6 |
| **166P** | 1 | 1 | 5 | 5 | 3 | 3 | 13 | 13 | 2 | 2 | 2 | 2 | 3 | 3 | 2 | 2 | 4 | 4 | 41 | 41 | ND | 8 | ND | 4 | 7 | 7 | 3 | 3 | 4 | 4 | 6 | 5 |
| **167P** | 1 | 1 | 5 | 5 | 3 | 3 | 13 | 13 | 2 | 2 | 2 | 2 | 3 | 3 | 3 | 2 | 4 | 4 | 41 | 41 | 8 | 8 | 4 | 4 | 7 | 7 | 3 | 3 | 4 | 4 | 5 | 5 |
| **168P** | 1 | 1 | 5 | 5 | 3 | 3 | 13 | 13 | 2 | 2 | 2 | 2 | 3 | 3 | 2 | 2 | 4 | 4 | 41 | 41 | ND | 8 | ND | 6 | ND | 7 | 3 | 3 | 4 | 4 | 5 | 5 |
| **169P** | **ND** | 3 | ND | 4 | ND | 3 | ND | 13 | 3 | 3 | ND | 2 | ND | 3 | 2 | 3 | 6 | 6 | 36 | 36 | 5 | 5 | ND | 2 | ND | 6 | ND | 3 | ND | 9 | 5 | 5 |
| **20Pa** | 1 | 1 | 5 | 5 | 3 | 3 | 13 | 13 | 2 | 2 | 2 | 2 | 3 | 3 | 2 | 2 | 4 | 4 | 41 | 41 | 8 | 8 | 6 | 6 | 7 | ND | 3 | 3 | 4 | 4 | 5 | 5 |
| **47Pa** | 1 | 1 | 5 | 5 | 3 | ND | 9 | ND | 2 | 2 | 2 | 2 | 3 | 3 | 2 | 2 | 6 | ND | 38 | 41 | 9 | 9 | 6 | 6 | 5 | 5 | 3 | 3 | 4 | 4 | 5 | 5 |
| **357Pa** | 1 | 1 | 5 | 5 | 3 | 3 | 13 | 13 | 2 | 2 | 2 | 2 | 3 | 3 | 2 | 2 | 4 | 4 | 41 | 41 | 8 | 8 | 10 | 10 | 7 | 7 | 3 | 3 | 5 | 5 | 5 | 5 |
| **463Pa** | 2 | 2 | 5 | 5 | 8 | 8 | 8 | 8 | 4 | 4 | 1 | 1 | 5 | 5 | 2 | 5 | 4 | ND | 38 | 38 | 9 | 9 | 5 | 5 | 4 | 4 | 9 | 9 | 2 | 2 | 6 | 6 |
| **770Pa** | 3 | 2 | 5 | 5 | 3 | 8 | 9 | 8 | 4 | 4 | 1 | 1 | 5 | 5 | 5 | 5 | 5 | 5 | 38 | 38 | 9 | 9 | 2 | 2 | ND | ND | ND | 9 | 2 | 2 | ND | 8 |
| **782Pa** | 3 | 3 | 4 | 4 | 3 | 3 | 13 | 13 | ND | ND | 2 | 2 | 3 | 3 | 3 | 3 | 6 | 6 | 36 | 36 | 6 | 5 | 2 | 2 | 5 | ND | 9 | ND | 3 | 3 | 5 | 5 |
| **804Pa** | 1 | 1 | 5 | 5 | 3 | 3 | 13 | 13 | 2 | 2 | 2 | 2 | 3 | 3 | 2 | 2 | 4 | 4 | 41 | 41 | 8 | 8 | 4 | 4 | 6 | 6 | 3 | 3 | 4 | 4 | 5 | 5 |
| **918Pa** | 2 | 2 | 5 | 5 | 3 | 8 | 9 | 8 | 3 | 3 | 1 | 1 | 3 | ND | 3 | 5 | 6 | 6 | 38 | 38 | 8 | 9 | ND | ND | 5 | 5 | 3 | 3 | 2 | 2 | 6 | 6 |
| **146-10RK** | 4 | 4 | 5 | 5 | 4 | 4 | 11 | 11 | 2 | 2 | 3 | ND | 3 | 3 | 3 | 3 | 6 | 6 | 43 | 43 | 8 | 8 | 3 | 3 | 7 | 7 | 3 | 3 | 3 | 3 | 5 | 5 |
| ***Brucella ovis*** |  |  |  |  |  |  |  |  |  |  |  |  |  |  |  |  |  |  |  |  |  |  |  |  |  |  |  |  |  |  |  |  |
| **183-7RK** | 2 | 2 | 5 | 5 | 8 | 8 | 9 | 8 | 4 | 4 | 1 | 1 | 5 | 5 | 5 | 5 | 6 | 6 | 38 | 38 | ND | 9 | ND | ND | 5 | 5 | 8 | 8 | 2 | 2 | 6 | 6 |
|  | **Bruce06** | | **Bruce08** | | **Bruce11** | | **Bruce12** | | **Bruce42** | | **Bruce43** | | **Bruce45** | | **Bruce55** | | **Bruce18** | | **Bruce19** | | **Bruce21** | | **Bruce04** | | **Bruce07** | | **Bruce09** | | **Bruce16** | | **Bruce30** | |
| **Strain** | **PCR - based MLVA** | **WGS - based MLVA** | **PCR - based MLVA** | **WGS - based MLVA** | **PCR - based MLVA** | **WGS - based MLVA** | **PCR - based MLVA** | **WGS - based MLVA** | **PCR - based MLVA** | **WGS - based MLVA** | **PCR - based MLVA** | **WGS - based MLVA** | **PCR - based MLVA** | **WGS - based MLVA** | **PCR - based MLVA** | **WGS - based MLVA** | **PCR - based MLVA** | **WGS - based MLVA** | **PCR - based MLVA** | **WGS - based MLVA** | **PCR - based MLVA** | **WGS - based MLVA** | **PCR - based MLVA** | **WGS - based MLVA** | **PCR - based MLVA** | **WGS - based MLVA** | **PCR - based MLVA** | **WGS - based MLVA** | **PCR - based MLVA** | **WGS - based MLVA** | **PCR - based MLVA** | **WGS - based MLVA** |
| ***Brucella suis*** |  |  |  |  |  |  |  |  |  |  |  |  |  |  |  |  |  |  |  |  |  |  |  |  |  |  |  |  |  |  |  |  |
| **183-6RK** | 2 | ND | 3 | 3 | 6 | ND | 10 | ND | 3 | 3 | 1 | 1 | 5 | ND | 2 | ND | 4 | 4 | 38 | 38 | 9 | 9 | ND | ND | 5 | ND | 10 | 10 | 5 | ND | 3 | 3 |
| **4Pa** | 2 | 2 | 5 | 5 | 8 | 8 | 9 | 8 | 4 | 4 | 1 | 1 | 5 | 5 | 5 | 5 | 6 | 6 | 38 | 38 | 9 | 9 | 8 | 8 | 5 | 5 | 4 | ND | 2 | 2 | 6 | 6 |
| **27Pa** | 2 | 2 | 5 | 5 | 8 | 8 | 8 | 8 | 4 | 4 | 1 | 1 | 5 | 5 | 5 | 5 | 6 | 6 | 38 | 38 | 9 | 9 | ND | ND | ND | 5 | ND | 11 | 2 | 2 | 8 | 8 |
| **183Pa** | 2 | 2 | 5 | 5 | 8 | 8 | 9 | 8 | 4 | 4 | 1 | 1 | 5 | 5 | 5 | 5 | 6 | 6 | 38 | 38 | 9 | 9 | ND | ND | 5 | 5 | 8 | 8 | 2 | 2 | 6 | 6 |
| **194Pa** | 2 | 2 | 5 | 5 | 8 | 8 | 9 | 8 | 4 | 4 | 1 | 1 | 5 | 5 | 5 | 5 | 7 | 7 | 36 | 38 | 9 | 9 | 11 | 11 | 4 | 4 | 13 | 13 | 2 | 2 | 6 | 6 |
| ***Brucella spp.*** |  |  |  |  |  |  |  |  |  |  |  |  |  |  |  |  |  |  |  |  |  |  |  |  |  |  |  |  |  |  |  |  |
| **MLVA22** | 1 | 2 | 3 | 3 | 3 | ND | 10 | 10 | 2 | 2 | 1 | 1 | 5 | 5 | 2 | 2 | 5 | 5 | 36 | 36 | 9 | 9 | 4 | 4 | 5 | 5 | 9 | 9 | 6 | 6 | 3 | 3 |
| **MLVA23** | 1 | 1 | 2 | 2 | 9 | 9 | 16 | 16 | 1 | 1 | 2 | 2 | 5 | 5 | 5 | 5 | 7 | 7 | 43 | 43 | 9 | 9 | 9 | 9 | 5 | 5 | ND | 3 | 9 | 9 | 5 | 5 |
| **MLVA24** | 2 | 4 | 5 | 5 | 4 | 4 | 11 | 11 | 2 | 2 | 2 | 2 | 3 | 3 | 3 | 3 | 5 | 5 | 43 | 43 | ND | 8 | 3 | 3 | 5 | 5 | ND | 3 | 4 | 4 | 5 | 5 |
| **MLVA25** | 2 | 2 | 4 | 3 | 6 | 6 | 9 | 9 | 3 | 3 | 1 | 1 | 5 | 5 | 2 | 2 | 4 | 4 | 38 | 38 | 9 | 9 | 5 | 6 | 6 | 6 | 5 | 5 | 5 | 5 | 3 | 3 |
| **MLVA31** | 1 | 4 | 5 | 5 | 4 | 4 | 13 | 12 | 3 | 3 | 3 | 3 | 5 | 5 | 1 | 1 | 6 | 6 | 41 | 41 | 9 | 9 | 5 | 3 | 6 | 6 | ND | 3 | 6 | 6 | 3 | 3 |
| **MLVA35** | 3 | 3 | 5 | 5 | 6 | 6 | 12 | 12 | 2 | 2 | 2 | 2 | 5 | 5 | 4 | 4 | 7 | 7 | 43 | 43 | 9 | 9 | 5 | 5 | 5 | 5 | 6 | 6 | 3 | 3 | 3 | 3 |
|  |  |  |  |  |  |  |  |  |  |  |  |  |  |  |  |  |  |  |  |  |  |  |  |  |  |  |  |  |  |  |  |  |
| **ND – not determined** | | |  |  |  |  |  |  |  |  |  |  |  |  |  |  |  |  |  |  |  |  |  |  |  |  |  |  |  |  |  |  |
